# Triclosan: An Update on Biochemical and Molecular Mechanisms

**DOI:** 10.1155/2019/1607304

**Published:** 2019-05-02

**Authors:** Mohammad A. Alfhili, Myon-Hee Lee

**Affiliations:** ^1^Department of Medicine (Division of Hematology/Oncology), Brody School of Medicine, East Carolina University, Greenville, NC 27834, USA; ^2^Department of Clinical Laboratory Sciences, College of Applied Medical Sciences, King Saud University, Riyadh 11433, Saudi Arabia

## Abstract

Triclosan (TCS) is a synthetic, chlorinated phenolic antimicrobial agent commonly used in commercial and healthcare products. Items made with TCS include soaps, deodorants, shampoos, cosmetics, textiles, plastics, surgical sutures, and prosthetics. A wealth of information obtained from *in vitro* and *in vivo* studies has demonstrated the therapeutic effects of TCS, particularly against inflammatory skin conditions. Nevertheless, extensive investigations on the molecular aspects of TCS action have identified numerous adversaries associated with the disinfectant including oxidative injury and influence of physiological lifespan and longevity. This review presents a summary of the biochemical alterations pertaining to TCS exposure, with special emphasis on the diverse molecular pathways responsive to TCS that have been elucidated during the present decade.

## 1. Introduction

Triclosan (TCS), or 5-chloro-2-(2,4-dichlorophenoxy)phenol, is a synthetic broad-spectrum antimicrobial developed in the 1960s. As a polychlorinated bisphenolic compound, TCS has a perceptible aromatic odor and is weakly soluble in water. It dissolves well in organic solvents including ethanol, dimethylsulfoxide (DMSO), and methanol [1], and the type of solvent and detergent availability seem to influence TCS activity [2–4]. For example, TCS dissolved in oils (e.g., olive oil) and alkali (e.g., sodium carbonate) exhibits markedly reduced efficacy when compared to other solvents such as glycerol and polyethylene glycol (PEG) [3, 5]. In fact, using propylene glycol (PG) as a solvent renders TCS more effective than using PEG, which is probably due to micellar solubilization of TCS in the larger PEG molecules [3]. Recently, we have shown that the presence of nonionic detergents (e.g., Tween 20) inhibits TCS activity *in vivo*, most likely due to micelle formation [6]. In contrast, sodium dodecyl sulfate (SDS) has been reported to potentiate the antibacterial effect of TCS *in vitro* [7].

TCS has gained enormous popularity in commerce and in healthcare owing to its antibacterial, antiviral, and antifungal properties [8–10]. This efficacy has led to the widespread use of TCS as a preservative in a variety of consumer products, including cosmetics, soaps, mouthwashes, antiperspirants, kitchen utensils, clothing textiles, bedclothes, electronics, plastics, and toys (Triclosan White Paper prepared by the Alliance for the Prudent Use of Antibiotics (APUA)). In clinical practice, TCS is used as a disinfectant and an antiseptic in surgical sutures, scrubs, implants, and medical devices [11, 12]. Annual global production of TCS was estimated at 1500 tons [13], and a total of 132 million liters of TCS-containing products was consumed in a single year in the United States (Safety and Effectiveness of Consumer Antiseptics; Topical Antimicrobial Drug Products for Over-the-Counter Human Use; Proposed Amendment of the Tentative Final Monograph. 2013 https://www.fda.gov/downloads/AboutFDA/ReportsManualsForms/Reports/EconomicAnalyses/UCM379555.pdf).

The high demand for TCS has consequently led to substantial buildup in drinking and wastewater sources and, more alarmingly, accumulation in body fluids [14–20], establishing the antimicrobial as an environmental pollutant. Pharmacokinetic studies in man show that TCS reaches the systemic circulation by rapid absorption through the skin and mucous membranes of the oral cavity and gastrointestinal tract, and variations in the bioavailability of TCS unsurprisingly affect the rate of urinary excretion [21, 22]. TCS content in commercial products may reach as high as 17 mM and comprise up to 1% of ingredients [12, 19, 23]. Moreover, absorption of up to 25% of applied TCS has been recorded [24], and metabolic studies in rats and mice revealed sulfation, glucuronidation, and hydroxylation products in tissues and excreta [25, 26].

Since the advent of TCS, early studies on the antiseptic have shown evidence of symptomatic relief from acne [27, 28] and contact dermatitis [29, 30] with fewer, or at least comparable, side effects to other therapeutic alternatives [31]. Later, TCS was found to be effective against crural ulcer [32] and chemically induced dermatitis and desquamation [33, 34], which could be attributed to its anti-inflammatory [35], hypoallergenic [36], and analgesic [37] properties. Moreover, a battery of studies collectively indicate that TCS is not a skin or oral mucosal irritant, has a very low sensitization potential (0.1-0.3% of 14,000 subjects), and is unlikely to be phototoxic to human skin (http://ec.europa.eu/health/ph_risk/committees/04_sccp/docs/sccp_o_166.pdf). This is in contrast to the reversible skin and eye irritation caused by up to 10% TCS reported in animals (http://ec.europa.eu/health/ph_risk/committees/04_sccp/docs/sccp_o_166.pdf). Also, in initial studies by Lyman and Furia, it was suggested that TCS is carcinogenic when orally administered to rats [38, 39]. Subsequent investigations in rats and mice disclosed that TCS perturbs microsomal detoxification [40], causes nephrotoxicity and hepatotoxicity [41], reduces prenatal and postnatal survival [42], and leads to central nervous system suppression [43] and hypothermia [44]. In humans, the earliest description of an adverse TCS reaction probably comes from a case report of two patients who developed contact dermatitis following application of deodorants containing 0.12% and 0.2% TCS [45]. Since then, several case reports of the same ailment have thus far been in congruence [46–49]. It is important to mention that, as is the case with healthy subjects, in patients diagnosed with, or suspected to have, contact dermatitis, TCS was similarly found to have a very low sensitization potential (0.6-0.8% of 11,887 patients) (http://ec.europa.eu/health/ph_risk/committees/04_sccp/docs/sccp_o_166.pdf).

In light of the dichotomous debate surrounding TCS, the US Food and Drug Administration (FDA), following extensive examination of available data, has effectively banned antiseptic products containing TCS since September 2016 [19]. In Europe, TCS was approved for use in cosmetics by the European Community Cosmetic Directive in 1986 (http://ec.europa.eu/health/ph_risk/committees/04_sccp/docs/sccp_o_166.pdf). However, the European Commission disapproved the use of TCS for hygienic purposes in 2017, but maintained its legality as a preservative in select cosmetics and mouthwashes in concentrations up to 0.3% and 0.2%, respectively (http://ec.europa.eu/health/scientific_committees/consumer_safety/docs/sccs_o_054.pdf; http://eur-lex.europa.eu/legal-content/EN/TXT/PDF/?uri=OJ:L:2014:107:FULL&from=EN). Furthermore, the Scientific Committee on Consumer Safety (SCCS) expressed its concern over the continued use of TCS in cosmetics, but not in antiseptics, mainly due to the cumulative pattern of exposure (http://ec.europa.eu/health/scientific_committees/consumer_safety/docs/sccs_o_054.pdf). Importantly, the European Chemicals Agency (ECHA) classifies TCS, under the classification, labeling, and packaging (CLP) regulation, as an eye irritant 2 (causes serious eye irritation), skin irritant 2 (causes skin irritation), aquatic acute 1 (very toxic to aquatic life), and aquatic chronic 1 (very toxic to aquatic life with long-lasting effects) (https://echa.europa.eu/documents/10162/21680461/bpc_opinion_triclosan_pt1_en.pdf/efc985e4-8802-4ebb-8245-29708747a358). Because of the previously mentioned ecotoxic properties, TCS is currently a candidate for substitution under the Biocides European Union regulation (Reg 528/2012/EC) (https://echa.europa.eu/potential-candidates-for-substitution-previous-consultations/-/substance-rev/12/term?_viewsubstances_WAR_echarevsubstanceportlet_SEARCH_CRITERIA_EC_NUMBER=222-182-2&_viewsubstances_WAR_echarevsubstanceportlet_DISS=true).

Our aim in this review is to provide an update on current knowledge regarding TCS therapeutic and toxic potential. Emphasis is placed on the biochemical and molecular alterations, either brought about by, or in response to, TCS exposure. Data from both *in vitro* and *in vivo* studies, obtained from humans and other organisms, are incorporated into the analysis, with special attention being given to reports published during the present decade.

## 2. Membrane and Cytoskeletal Damage

Perhaps the earliest report describing the antimicrobial activity of TCS was by Vischer and Regös [50] which was shown through topical application. In a follow-up study, TCS was found to be more effective with the broadest spectrum against bacteria and fungi when compared to other antimicrobials such as gentamicin and clotrimazole [10]. Subsequent efforts, which continue to this day, have focused on dissecting the diverse action mechanisms and cellular targets of TCS. Initially, it was thought that TCS interacts with the prokaryotic cell membrane nonspecifically [9]. This was corroborated by the resistance of Gram-negative bacteria to TCS, which was ascribed to their outer membrane [51, 52]. Investigating the genetic response of *Mycobacterium tuberculosis* to TCS, Betts et al. [53] identified perturbations in a wide assortment of genes involved in cell wall, transport, detoxification, and DNA replication and transcription. Also, *Klebsiella pneumoniae* with inactive efflux pump *KpnGH* exhibit pronounced susceptibility to multiple antibiotics including TCS [54]. Several genes in the membrane stress response pathway were also studied in *Escherichia coli* and *Rhodospirillum rubrum* S1H [55–57]. During the electro-Fenton transformation of TCS, significant changes in expression patterns of genes involved in cell wall and membrane structure, cell envelope, flagella, and multidrug efflux were observed (Table 1). These findings complement an earlier report describing enhanced resistance to TCS due to overexpressed *acrAB* multidrug efflux pump [58]. It was recently suggested that TCS binds to the transcriptional repressor AcrR, causing conformational changes and preventing its binding to the efflux pump *AcrA* promoter in *Agrobacterium tumefaciens* [59].

The interaction of TCS with the cell membrane was also studied in human red blood cells (RBCs; erythrocytes). TCS exposure led to K^+^ leakage and overt hemolysis, indicating membrane damage, while antagonizing hypotonic lysis, which may be due to membrane expansion [60]. TCS also inhibited membrane-bound Na^+^,K^+^,Mg^2+^-ATPase enzymatic activity [61]. These observations suggest that TCS causes membrane destabilization, perturbs monovalent ion transport, and modulates the overall osmoregulation of erythrocytes. Evidence for membrane damage is further confirmed in numerous studies by means of compromised stability and permeability [62]. To directly observe how TCS interacts with the cell membrane, Guillén and coworkers utilized nuclear magnetic resonance (NMR) spectroscopy to demonstrate that TCS intercalates within hydrophobic pockets in the lipid bilayer, perpendicularly to phospholipid molecules [63]. Furthermore, using neutral red to evaluate membrane integrity, diminished uptake of the dye in hemocytes of the clam *Ruditapes philippinarum* and mussel *Mytilus galloprovincialis* was related to TCS-induced suppression of pinocytosis and disturbed phagocytosis [64, 65].

Along those lines, our recent findings indicate that TCS blunts the expression of the *pmp3* membrane transporter in *Caenorhabditis elegans* nematodes and that *pmp3(ok1087)* mutants exhibit increased sensitivity to the disinfectant [66]. Finally, a proteomic analysis of zebrafish (*Danio rerio*) larvae and gills of freshwater mussel *Dreissena polymorpha* revealed alterations in cytoskeletal protein levels following TCS exposure (summarized in Table 1) [67, 68].

There is a consensus in the literature regarding the membranotropic nature of TCS in different membrane models across various species. The cell membrane is a primary target for TCS and among the first cellular obstacles that must be overcome by the antiseptic to exert its effects. Although evidence implicating membrane-associated efflux pumps as part of the cellular response to TCS is strong, there is paucity in reports describing TCS modulation of structural or functional membrane components in human-based systems. Similarly lacking is an understanding of the role of membrane receptors not only in pumping out TCS molecules but also in transducing both inter- and intracellular signals as a consequence to TCS presence.

## 3. Cellular Longevity

The interest in TCS and ultimate cell fate has originally stemmed from its use in oral hygiene products, which is reflected in two seminal studies on human gingival cells [69, 70]. TCS was shown to be cytotoxic to gingival fibroblasts and epithelial cells, identifying it as a novel stimulator of apoptosis in the latter.

Investigations have thus far followed a more comprehensive approach, relating cell death induced by TCS to other cellular adversaries, utilizing both human and non-human model systems. When TCS was treated to human choriocarcinoma placental cells (JEG-3), multiple dose- and time-dependent responses were observed [71]. While there was a proportional increase in estradiol and progesterone secretion, *β*-human chorionic gonadotropin (*β*-hCG) release was nevertheless inhibited with increasing TCS concentrations [71]. In addition to blunted proliferation, significant cell death was recognized as apoptotic in nature evidenced by activated caspase-3 and Hoechst 33342-stained fragmented DNA [71]. Similarly, using anoikis-resistant H460 human lung cancer cells, Winitthana et al. demonstrated that 24-hour exposure to 10 *μ*M TCS causes cell death and apoptosis. Nontoxic levels (≤7.5 *μ*M), however, enhanced cell growth (increased colony number and reduced size) without altering proliferation. TCS also promoted epithelial-to-mesenchymal transition (EMT), along with the migratory and invasive abilities of the cells [72].

A research group performed a series of *in vivo* and *in vitro* studies on the effect of TCS on growth and proliferation of human BG-1 ovarian cancer cells. Results from these studies indicate that TCS increases cellular proliferation and both gene expression and protein levels of cyclin D1 and decreases p21 and Bax gene expression and protein levels [73]. These effects were significantly antagonized by the estrogen receptor (ER) antagonist ICI 182,780, implicating ER in TCS-induced cell cycle progression and in its antiapoptotic role. Investigators from the same group also reported a similar response to TCS by MCF-7 breast cancer cells and LNCaP prostate cancer cells. In MCF-7 cells, 1 *μ*M TCS enhanced growth and proliferation during a six-day period, which was associated with increased cyclin D1 and reduced p21 expression levels. When mice were treated with TCS for 8 weeks, brdU-positive breast tumor cells were significantly increased compared to the control group treated with corn oil [74]. Similar to BG-1 cells, TCS-promoted proliferation of MCF-7 cells was mediated through ER*α* signaling, demonstrated as antagonism by kaempferol and 3,3′-diindolylmethane (DIM), two phytoestrogens [75]. In addition to cyclin D1 and p21, TCS caused an increase in cyclin E and a decrease in Bax and induced metastasis through elevated cathepsin D protein expression. These observations were paralleled *in vivo* using xenografted mouse models. Researchers from this report expanded their findings to VM7Luc4E2 cells, a variant of the MCF-7 model, to show that TCS (0.1-10 *μ*M) is pro-proliferative and antiapoptotic by inhibiting oxidative stress, with both effects being antagonized by kaempferol [76]. In LNCaP cells exposed to concentrations of TCS ranging from 0.01 to 10 *μ*M for up to 5 days showed enhanced proliferation and migration and reduced p21 protein expression [77]. In primary human syncytiotrophoblasts, TCS at 0.001 to 10 *μ*M induced apoptosis as seen by condensed nuclei and fragmented DNA [78]. TCS also reduced 11*β*-hydroxysteroid dehydrogenase type 2 (11*β*-HSD2) via a caspase-dependent mechanism. Other targets included both Bax and Bcl-2 proteins.

Similar to human cells, both pro- and antiapoptotic properties were observed in rodent cells treated with TCS. Beside its cytotoxicity, TCS caused caspase-dependent apoptosis in rat neural stem cells along with elevated Bax and reduced Bcl-2 [79]. In a series of studies, [80–82] mouse neurons were used to show that TCS is apoptotic through the Fas receptor (FasR), aryl hydrocarbon receptor (AhR), and caspase activation involving N-methyl-D-aspartate receptors (NMDARs). In agreement with the cytotoxicity data, TCS-treated mouse lung epithelial cells were deformed with reduced viability [83]. Conversely, TCS stimulated the proliferation of mouse epidermis-derived JB6 Cl 41-5a cells, by increasing cyclins D1 and A and reducing p27(Kip1) protein levels [84]. Examining these effects *in vivo*, B6C3F1 mice exhibited epidermal hyperplasia and focal necrosis following topical administration of TCS. Moreover, the pluripotency markers of mouse embryonic stem cells were analyzed following TCS exposure [85]. Alkaline phosphatase (*Alp*), *Sox2*, *Oct4*, and *Nanog* were all reduced, while miRNA-134 was elevated.

Unlike human and rodent cells, *in vivo* and *in vitro* studies on aquatic organisms uniformly agree that TCS is solely proapoptotic in these animals. Pyknotic apoptosis in the central nervous system of zebrafish *D. rerio* was observed following treatment with either TCS alone or TCS combined with derivatives 2,4,6-trichlorophenol (2,4,6-TCP) and 2,4-dichlorophenol (2,4-DCP) [86, 87]. The TCS-derivative mixture caused pronounced deformities and behavioral abnormalities and perturbed the expression of a panel of neurodevelopmental and apoptotic genes (Table 2). Also, TCS, following both *in vivo* and *in vitro* exposure, induced a dose- and time-dependent increase in apoptotic hemocytes of *D. polymorpha* [88, 89]. Likewise, when the saltwater clam *Ruditapes philippinarum* was treated with TCS, hemocytes exhibited significant cell death, blunted proliferation, reduced size, and promiment apoptotic DNA fragmentation [65]. TCS-induced apoptosis, or apoptosis-like cell death, was also detected in unicellular organisms, such as the green alga *Chlamydomonas reinhardtii* and the pathogenic fungus *Cryptococcus neoformans* [90, 91].

Collectively, studies on TCS influence on cell fate indicate estrogenic, proliferative, and apoptotic activities. Genes and proteins governing the regulation of cell cycle and apoptosis are particularly sensitive to TCS modulation. The disparity in ultimate cell fate seems to point at an interspecies variation and a dose-specific response, among other experimental details such as cell type and duration of exposure. Elucidating the existence and the identity of a specific molecular “switch” that may tip the scales in favor of either cell death or survival could be an important inquiry for future investigations.

## 4. Oxidative Stress

Overwhelming evidence has recently accumulated in support of the prooxidative action of TCS. It is prudent to provide an overview of human-based studies first before summarizing notable findings obtained from other model organisms.

In Puerto Rican pregnant women, a correlation between exposure to TCS during pregnancy and oxidative damage, as measured by urinary 8-hydroxyguanosine (8-OHdG), and inflammation was suggested [92]. Similar observations were also mirrored in Chinese and Brazilian children [93, 94]. Conversely, in a global effort comprising nine countries from Asia, Europe, and North America, no relation between urinary TCS and 8-OHdG was established [95].

*In vitro* studies on human cells have also shed some light on the oxidative potential of TCS. In peripheral blood mononuclear cells (PBMC), 2,4-dichlorophenol (2,4-DCP)—a product of TCS transformation—promoted reactive oxygen species (ROS) generation, with subsequent lipid peroxidation and protein carbonylation [96]. Similarly, TCS caused elevated ROS in Nthy-ori 3-1 human follicular thyroid cells [97] and lipid peroxidation in retinoblastoma (Y79 RB) cells [98]. Our recent investigations on mesenchymal stem cells also showed TCS interference with the activation of nuclear factor (erythroid-derived 2)-like 2 (Nrf2), the “master regulator” of detoxification, and its downstream targets, heme oxygenase 1 (HO-1) and NAD(P)H dehydrogenase [quinone 1] (NQO-1) [66]. Consistently, TCS incorporated in mouthrinse did not exhibit antioxidant activity on fibroblasts [99]. In contrast, TCS reduced ROS levels in VM7Luc4E2 cells, which contributed to its antiapoptotic activity in these malignant breast cells [76].

Mitochondrial damage was also evident in multiple mammalian cells including human PBMC and keratinocytes, exposed to 3.5-350 *μ*M TCS [100]. At concentrations up to 100 *μ*M, TCS caused depolarization of mitochondrial membrane, reduced oxidative phosphorylation, and suppressed ATP synthesis. Weatherly et al. [101] utilized human HMC-1.2 mast cells and primary keratinocytes to show that TCS is a proton ionophore uncoupler and interferes with ATP production.

Animal studies conducted on mice and rats have revealed a profound response in the cellular antioxidant machinery upon TCS treatment. In rat thymocytes, superoxide anions were found to be elevated following TCS treatment [102] which, as Yueh et al. [103] showed, was met with increased expression of key antioxidant enzymes including HO-1, NQO-1, and glutathione S-transferase (GST) in mouse liver. Evidence for testicular DNA damage, elevated malondialdehyde (MDA), and superoxide dismutase (SOD), in addition to diminished catalase (CAT), was related to TCS treatment in weanling rats [104]. Similarly, in lung homogenates of female albino rats, TCS was found to induce lipid peroxidation and severely deplete the levels of other crucial antioxidants: SOD, CAT, and glutathione (GSH) [105]. Increased expression of glutathione peroxidase 1 (*Gpx1*) and aldehyde oxidase 1 (*Aox1*) was also observed as a consequence to TCS exposure in C57BL/6 mice [106]. Most recently, Zhang et al. [97] showed downregulation of antioxidant enzymes, *Gpx3*, *Cat*, and *Sod2*, along with elevated MDA, in the hypothalamus of Sprague-Dawley rats. Moreover, it was found that TCS treatment leads to increased ROS and reduced GSH activity in rat neural stem cells [79]. TCS also increased ROS levels in mouse neocortical neurons, along with perturbed regulation of cytochrome P450 family 1, subfamily a, member 1 (CYP1a1) and CYP1b1 [81, 82]. Effects of TCS on cytochromes and hepatic detoxification were also demonstrated in Sprague-Dawley rats, showing increased levels of UDP-glucuronosyltransferase 1-1 (Ugt1a), Ugt2b1, CYP1a1, CYP1a2, CYP2b1, CYP3a1, and sulfotransferase family 1E member 1 (Sult1e1) [97].

Several terrestrial organisms have been employed in the study of TCS toxicology. *Caenorhabditis elegans* is among the best-studied animal models due to its ease of maintenance and high genetic homology to humans. We have recently shown that TCS leads to overproduction of ROS, inhibition of nuclear translocation of protein skinhead-1 (SKN-1) antioxidant transcription factor, and downregulation of gamma-glutamyl cysteine synthetase (*Gcs1*) [66]. In a subsequent report, *Skn1* expression was found to be upregulated by TCS along with *Sod1*, *Sod4*, heat shock proteins (*Hsp*)*-3*, *-4*, *-16.2*, and *-70*; and cytochromes *Cyp29A2* and *Cyp34A9* (https://app.dimensions.ai/details/publication/pub.1103154992#readcube-epdf). TCS also enhanced nuclear translocation of stress-related factor DAF-16, suggesting the occurrence of oxidative stress [107]. In the Earthworm *Eisenia fetida*, oxidative damage by TCS was manifested as a transient elevation in CAT and GST enzymes, increased MDA, and DNA damage [108]. In a follow-up study by the same group, SOD was also increased and decreased by TCS depending on the concentration used [109], a response mirrored by CAT in the snail *Achatina fulica* [110]. In that study, TCS caused diminished levels of SOD and peroxidase (POD), along with elevated MDA, among other morphological anomalies.

The ubiquity of TCS in aquatic environments has made animal models from that habitat the subject of extensive investigations on TCS toxicity. Perhaps the most relevant aquatic organism is the zebrafish *D. rerio*, owing to a strong structural and molecular resemblance to humans. Elucidating the interaction between TCS and the antioxidant system in ZFL liver cells, Zhou et al. [111] showed evidence of induced CYP1A activity along with a general trend of suppression in phase I and II detoxification enzymes. Elevated MDA, along with perturbed homeostasis of GSH, peroxiredoxin-2 (PRD-2), and HSPs, were observed in zebrafish larvae grown in the presence of TCS ([67, 87]).

TCS has been shown to induce MDA and cause oscillations in CAT, ethoxyresorufin-O-deethylase (EROD), erythromycin N-demethylase (ERND), and aminopyrine N-demethylase (APND) in *Daphnia magna* [112]. Moreover, elevated amino acids, including glutamine, glutamate, and proline, have been attributed to a general oxidative stress state in daphnids [113]. Also, stress-related proteins, including glyceraldehyde 3-phosphate dehydrogenase (GAPDH) and hsp-70, were modulated by TCS in *D. polymorpha*, in addition to lipid peroxidation [68]. TCS exposure demonstrated reduced oxyradicals and lipofuscin and elevated oxidized glutathione (GSSG) in the digestive gland of swollen river mussels *Unio tumidus* [114]. In *Tigriopus japonicus* copepods treated with TCS, increased ROS, SOD, GST, GPx, and GSH content was noted [115]. TCS also caused perturbations in expressional profiles of *Cyps*, *Sod*, *Gst*, and *Cat* proteins (Table 3) [115].

TCS treatment in the yellow catfish *Pelteobagrus fulvidraco* revealed induced CAT, EROD, ERND, and APND [116]. Expressional profiling of *Cyp1a*, *Cyp3a*, and *Gst* showed both up- and downregulation depending on TCS concentration and length of exposure, a pattern that was also seen with MDA formation. When another catfish, *Heteropneustes fossilis*, was treated with a cosmetic effluent rich in TCS, increased SOD and CAT activities and reduced GSH, GST, and GPx were noted [117].

Oxidative damage by TCS was also evident in the goldfish *Carassius auratus*, as MDA, CAT, and GSH were elevated in addition to a reduced total antioxidant capacity [118]. Variable responses by antioxidant enzymes and in MDA levels were recorded in the goldfish's liver after TCS treatment under a pH range of 6 to 9 [119]. The oxidative potential of TCS was also evident in the rotifer *Brachionus koreanus*, detected as ROS overproduction and enhanced GST activity, in addition to transcriptional modulation of cytochromes, antioxidant genes *Gst*, *Gpx*, *Sod*, and *Cat* and chaperons (Table 3) [120]. Moreover, TCS inhibited *Sod* and phospholipid hydroperoxide glutathione peroxidase (*Phgpx*) expression in the liver of *Bufo gargarizans* tadpoles [121] and induced GST in *Pelophylax perezi* frog larvae [122].

Sendra et al. [123] studied the combined effect of titanium dioxide (TiO_2_) and a heterogeneous mixture of organic compounds including TCS using the clam *Ruditapes philippinarum*. Modulations in EROD, SOD, CAT, GPx, GST, and GR enzyme activities were noted in the clam's digestive gland, in parallel with increased lipid peroxidation. TCS exposure caused alterations in *Cat*, *Sod*, *Gpx1*, *Gpx2*, *Gsta*, *Hsp90bb*, *Hsp90ba*, and *Hsc70a* genes in rainbow trout *Oncorhynchus mykiss* [124]. Although in one report TCS failed to elicit oxidative stress in the green algae *Chlamydomonas reinhardtii* [125], another report detected ROS formation following TCS exposure [90], which was also most recently confirmed by significantly increased MDA, downregulated *Gpx*, and upregulated *Sod* expression [126].

The antimicrobial nature of TCS makes bacteria an appropriate target for mechanistic studies. Using *Rhodospirillum rubrum* S1H, Pycke et al. [57] detected upregulation in a host of TCS-induced oxidative response genes, most notably *Gpx*. In *E. coli* K12, MG1655, the electro-Fenton transformation of TCS caused activation of genes related to ROS sensing, along with reduced glutaredoxin (*Grx*), *Sod*, *Cat*, and alkyl hydroperoxide reductase (*Ahpr*) [55]. Very recently, ROS formation by TCS was associated with diminished expression of antioxidants in *E. coli* (Table 3), an event that preceded mutagenesis and enhanced drug resistance in that species [56]. TCS was also recently used to validate novel self-luminescent bioreporter strains of *Nostoc* sp. PCC 7120 using *Sod* promoters [127].

Collectively, monumental evidence demonstrates the prooxidant properties of TCS evident as both overproduction of ROS and interference with the cellular antioxidant defensome. TCS is toxic in part by inducing oxidative damage in a wide range of organisms and by targeting a defined cluster of proteins in a fashion that is conserved among diverse species. Nonetheless, the vast majority of data are collected from non-human models, and, as is the case with other toxicological reports of TCS, studies conducted on man or human-derived tissues are severely lacking.

## 5. Immunity and Inflammation

TCS has, for a long time, been recognized as an effective therapy for infectious dermatitis [29–31], and the observed curative capacity of the compound was solely attributed to its antimicrobial activity. It was not until the end of last century that associations between TCS exposure and remission of noninfectious inflammation were made [33, 35, 36], and the use of antibacterials as anti-inflammatory agents has gained deserved attention during the past two decades. For example, an appreciable number of antibiotics, including macrolides and quinolones, have been shown to possess anti-inflammatory activity [128–132]. Follow-up efforts have successfully provided solid evidence for the direct interaction of TCS with inflammatory pathways.

Gaffar et al. [133] reported that TCS inhibits cyclooxygenase-1 (COX-1) and COX-2, 5-lipoxygenase and (LPO), 15-LPO, and interleukin- (IL-) 1*β*-induced prostaglandin E2 (PGE2) in gingival cells. TCS was also shown to suppress a wider range of inflammatory mediators including IL-1*β*-induced prostaglandin I2 (PGI2) and arachidonic acid, tumor necrosis factor (TNF)*α*-induced PGE2, phospholipase A2 (PLA2), and COX [134]. Moreover, in a double-blind crossover study, participants who used a mouthrinse with added 0.15% TCS developed significantly less oral erythematous lesions than those who used a TCS-free mouthrinse [135]. By then, the anti-inflammatory properties of TCS were established and were widely accepted within the scientific and medical communities.

TCS in prosthetic devices was found to have no influence on the acute phase response [136], and only modest differences were seen between TCS and stannous fluoride dentrifice [137]. Nevertheless, TCS, when applied intracrevicularly, improved clinical parameters of gingivitis [138]. In a recent double-blind, randomized, crossover study, it was concluded that TCS-containing toothpaste inhibits inflammation in peri-implant tissue [139].

To date, elaborations on the anti-inflammatory nature of TCS have been the focus of subsequent studies. Mustafa et al. [140–142] identified IL-1*β*, interferon (IFN)*γ*, major histocompatibility complex (MHC) class II, and PGE synthase-1, as targets of TCS in human gingival fibroblasts. Of note, studies to discern the subcellular localization of TCS show preference for nuclear, as opposed to cytosolic, accumulation. Although initial uptake was considerably higher in the cytoplasm, a great proportion of cytosolic TCS was eliminated after repeated washing, while nuclear retention was observed [143]. This may explain the perturbed inflammatory signaling associated with TCS. Moreover, in primary human oral epithelial cells, TCS attenuated LPS-induced cytokine response including IL-8, IL-1*α*, and TNF*α* and aggravated the antimicrobial response, which was mediated through microRNA (miRNA) regulation of the toll-like receptor (TLR) pathway [144]. The findings were also reciprocated in cells derived from diabetic patients, with an exaggerated TLR response [145]. It was revealed that TCS, nevertheless, abrogated LPS-induced TLR response, again, through regulating miRNAs (stimulating miR146a and inhibiting miR155s).

In skin and leukocytes of mice topically treated with TCS, alterations in inflammatory responses were mediated through TLR4 [146]. Likewise, TCS downregulated parathyroid hormone- (PTH-) or PGE2-stimulated matrix metalloproteinase-13 (MMP-13) expression in rat osteoblastic osteosarcoma cells [147]. Since hyperactive MMP-13 is implicated in periodontal disease, it was suggested that TCS might have a protective role against oral inflammatory conditions through its action on that enzyme, among others [148].

Interestingly, favorable results have been observed for TCS against other inflammatory conditions including cardiovascular disease and hidradenitis suppurativa [149, 150]. Moreover, the use of TCS-impregnated ureteral stents seems to be a promising approach to combat urinary tract infections (UTI) and associated inflammation [151, 152]. Along those lines, an increased urinary TCS was related to increased serum IL-6 in pregnant women [92], pointing at a possible pro- or anti-inflammatory role.

In a unique effort by Barros et al. [153], TCS modulation of the inflammatory response in an ex vivo whole blood stimulation assay was investigated. In that study, TCS inhibited multiple inflammatory mediators induced by LPS, including interleukins, most notably IL-1 & IL-6, IFNs, and colony-stimulating factor (CSF) 2. Activation of type 1 T helper lymphocytes was interrupted through the action of TCS on CD70. In a related report, TCS also reduced the capacity of natural killer (NK) lymphocytes to lyse chronic myelogenous leukemia K562 cells [154]. Recently, chitosan-TCS particles reduced the expression of IL-1*β*-induced *Cox2* and *Il6*, among other immune molecules in gingival fibroblasts (Table 4) [155], showcasing the vast amenability of this antimicrobial to nanoparticle manipulation.

Other *in vivo* studies on rodents and marine organisms clarified further the immunomodulatory properties of TCS. For instance, in mice subjected to an acute, systemic *E. coli* infection, Sharma et al. [156] demonstrated that cotreatment with TCS significantly reversed the damage caused by the bacteria. Specifically, TCS prolonged survival; lessened hepatic congestion, hemorrhage, and fatty changes; and reduced blood liver enzymes, serum TNF*α*, and the severity of bacteremia. In accordance with published data, TCS was similarly immunosuppressive in aquatic mussels (*M. galloprovincialis*) and clams (*R. philippinarum*) [64, 65].

Contrary to the overwhelming evidence of the anti-inflammatory function of TCS, a number of studies have nonetheless identified a proinflammatory role by the antiseptic. For example, upon intratracheal instillation of TCS in Sprague-Dawley rats, elevated total cell (TC) count, polymorphonuclear leukocytes (PMNs), total protein (TP), LDH, TNF*α*, and IL-6 were observed in bronchoalveolar lavage (BAL) fluid [83], which, except for TP, returned to baseline levels 14 days after exposure. Consonantly, it has also been demonstrated that TCS exacerbates diethylnitrosamine-induced hepatocellular carcinoma in C57BL/6 mice [103]. Likewise, TCS was very recently found to increase *Tlr4* expression to promote colitis and aggravate colitis-related cancer in C57BL/6 mice [157].

It is evident from the wealth of information present that TCS is a modulator of immune and inflammatory reactions. The sum of data from *in vitro* and *in vivo* studies indicates that TCS, on its own, is immunosuppressive. Nevertheless, increasing evidence seems to suggest that in the presence of an existing adverse condition, such as inflammation or tumor, TCS further potentiates and worsens the eventual outcome. Investigations into the molecular basis behind this unique behavior are particularly warranted.

## 6. Genotoxicity and Carcinogenicity

Among the most important aspects of toxicological profiling of compounds is their interaction with the molecule of life—the DNA. Early efforts [42, 158] point at a possible role for TCS in somatic mutations observed in mice. TCS also caused a significant reduction in global DNA methylation in human hepatocellular carcinoma HepG2 cells, a finding associated with liver tumor [159]. Similarly, TCS caused a dose-responsive increase in chromosomal aberrations in lung fibroblast V79 cells, but not in ovary CHO cells, of the Chinese hamster *Cricetulus griseus* [12]. In a comparative study on *Drosophila melanogaster* using three mouthwashes, namely, Cepacol® (0.05% cetylpyridinium chloride), Periogard® (0.12% chlorhexidine digluconate), and Plax® (0.03% TCS), it was concluded that only the ethanol content in Cepacol®, but not other active ingredients, caused mitotic recombination between homologous chromosomes [160]. On the other hand, TCS induced dose-responsive DNA damage in hemocytes of the zebra mussel *D. polymorpha* [88], and strand breaks in the digestive gland of *U. tumidus* mussels [114]. A similar dose-dependent DNA damage was also observed in the earthworm *E. fetida* [108, 109], but not in *E. andrei* [161].

Comparing TCS to other toxicants in the larvae of freshwater insect *Chironomus riparius*, Martinez-Paz et al. [162] found TCS, along with nonylphenol, to be the most potent in causing DNA breakage. It was also noted that TCS, either alone or in combination with carbendazim, induced DNA damage in *D. magna* [163]. Using the brine shrimp *Artemia salina*, a time-dependent pattern of TCS-induced genotoxicity was identified [164]. Moreover, TCS was genotoxic in the catfish *Heteropneustes fossilis*, goldfish *C. auratus*, and rainbow trout *O. mykiss* [117, 118, 124]. Importantly, when TCS at an environmentally relevant concentration (3 nM) was treated to the freshwater protozoan *Tetrahymena thermophila*, notable DNA damage, without significant perturbation in growth or cell viability, was evident [165]. In a more detailed study on *E. coli*, Gou et al. [55] revealed that the electro-Fenton transformation of TCS caused upregulation of a host of genes involved in the DNA repair machinery, indicative of DNA stress. These genes belong to base excision repair (*mutT* and *nfo*), nucleotide excision repair (*uvrA* and *uvrD*), mismatch repair (*uvrD* and *ssb*), and double-strand break repair (*ssb* and *recN*). Chromosomal stickiness, reduced mitotic activity, and ana-telophase bridges were also noticeable in the bulb onion *Allium cepa* following TCS treatment [166].

In a recent proof-of-concept study, the promising potential of a toxicogenomic approach as a follow-up to positive *in vitro* genotoxicity data was evaluated. Using TCS as a testing compound, it was shown that the antimicrobial is non-DNA reactive and that it is genotoxic solely *in vitro* as opposed to *in vivo* [167].

Ambiguity surrounding the carcinogenicity of TCS still remains today. Investigators have generally been able to provide evidence for carcinogenic effects in animal models but not in humans. Of the earliest studies in this regard was a report by Lyman and Furia [38] identifying TCS as a carcinogen in mice. Other studies on mice have been in agreement with that conclusion. For example, it was noted that chronic TCS exposure increased the incidence of liver neoplasms [12] and aggravated hepatocellular carcinoma [103]. Furthermore, TCS caused colonic inflammation and worsened colitis or tumorigenesis induced by dextran sodium sulfate [168]. These findings, were, however, not paralleled in rats, hamsters, or baboons [12, 169]. More importantly, *in vivo* human studies of TCS are scarce, and aspects related to TCS-induced oncogenesis are lacking. Consequently, whether TCS poses a carcinogenic hazard to humans is unknown and requires further investigation. Nonetheless, the interaction of TCS with human-derived cancer cells *in vitro* has recently gained considerable attention (reviewed under Therapeutic Proposals).

In light of available data (Table 5), TCS demonstrates carcinogenicity solely in mice and within a narrow range of tissues (the liver and colon), which constitutes limited evidence of carcinogenicity according to ECHA (https://echa.europa.eu/documents/10162/23036412/clp_en.pdf/58b5dc6d-ac2a-4910-9702-e9e1f5051cc5). Hence, TCS is not classifiable as a carcinogen (http://ec.europa.eu/health/scientific_committees/consumer_safety/docs/sccs_o_054.pdf). It must be noted that in case future assessment conclusively rules out TCS as a human carcinogen, caution with its use must still be exercised given the established carcinogenicity of its transformation products—dioxins, chloroform, and anilines [170].

## 7. Cellular Signaling

Adaptations to the ever-changing intracellular and surrounding environments are achieved, in large part, by effective communication. Transmission of information that carries specific instructions is executed by messengers that function in tandem within a defined pathway. Tasks, however, are usually accomplished through the sequential transduction of multiple messages along a complex, intertwining network that involves a wide assortment of mediators [171]. Hence, the participation of cell signaling cascades in the response to xenobiotics cannot be overlooked.

The use of human cell lines has provided a wealth of information particularly regarding the study of signaling molecules responsive to stressors and xenobiotics, including TCS. In H460 lung cancer cells, TCS promoted migration and invasion through focal adhesion kinase/ATP-dependent tyrosine kinase (FAK/Akt) and Ras-related C3 botulinum toxin substrate 1 (Rac1) [72]. Evidence similarly exists for the classical mitogen-activated protein kinases (MAPK) as targets of TCS. For example, proliferation of JB6 Cl 41-5a cells as induced by TCS was accompanied by activation of extracellular signal-regulated kinases 1/2 (ERK1/2), c-Jun N-terminal kinases (JNK), and p38 MAPKs, in addition to Akt [84]. Importantly, blocking either MEK1/2 or phosphoinositide 3-kinase (PI3K) significantly attenuated TCS-induced proliferation. In another study on rat neural stem cells, TCS-induced cytotoxicity and apoptosis were accompanied by activation of p38 and JNK and suppression of ERK, Akt, and PI3K [79]. This points at the involvement of these proteins in both cellular survival and death as brought about by TCS. Recently, TCS was shown to activate p38 and JNK *in vivo* as detected in the hypothalamus of Sprague-Dawley rats and *in vitro* utilizing human Nthy-ori 3-1 thyroid follicular cells [97]. In that study, TCS stimulated the thyrotropin-releasing hormone receptor through p38 MAPK, which, in turn, influenced the thyroid peroxidase (TPO) level.

In suppressing TLR signaling in whole blood leukocytes, TCS downregulated the expression of several signaling mediators, most notably, NF-*κ*B-inducing kinase (*Nik*) and *C-jun*, which accounted for the overall blunted inflammatory response to LPS in these cells [153]. Furthermore, suppression of *Mmp-13* expression in mouse osteoblastic osteocarcinoma cells by TCS was possibly related to its inhibition of Fos/Jun and AP-1 sequence binding in both the *Mmp-13* and *C-fos* promoters [147].

The endocrine-disrupting activity of TCS, specifically its estrogenicity, has been of great interest to researchers. Kim et al. [73] utilized BG-1 ovarian cancer cells to show that the proliferative effects of TCS were mediated through ER*α*. Confirming the ER's role, the use of ICI 182,780 reversed the proliferative properties of TCS along with associated perturbations in cyclin D1, p21, and Bax expression and protein levels. Likewise, the ER is implicated in TCS-induced proliferation of MCF-7 cells and increased breast tumor mass in mice [74, 75, 172]. This was similarly indicated by TCS inhibition with ICI 182,780 or kaempferol and the stimulation of insulin-like growth factor (IGF) signaling, namely, phosphorylated insulin receptor substrate (pIRS-1), pAkt, pMEK1/2, and pERK1/2 [75]. Notably, kaempferol also inhibited TCS-induced VM7Luc4E2 cell growth [76]. These observations are in congruence with an earlier report by Huang et al. [173] describing the estrogenic activities of nanomolar concentrations of TCS in the same cells. Investigating ER-responsive genes on the transcriptional and translational levels, it was shown that TCS induced pS2 but blunted ER*α* mRNA and protein levels, the latter of which was related to elevated miR-22, miR-206, and miR-193b miRNAs.

Recent studies have also argued for the dual effect of TCS on ER signaling. For example, Henry and Fair [174] demonstrated that, when administered alone to MCF7 cells, TCS at 7 nM to 700 *μ*M exhibits estrogenic activity but becomes antiestrogenic in the presence of E2. Along those lines, it was shown that TCS, on its own, lacked any effect on rat uterine growth, but could still potentiate the effect of ethinylestradiol (EE) [175]. In a follow-up investigation, it was reported that TCS promotes EE-induced inhibition of ER*α* and ER*β* expression and when given alone does not activate ER at concentrations from 30 nM to 100 *μ*M [176]. Furthermore, TCS diminished E2 and estrogen sulfotransferase in sheep placenta [177]. This is in contrast to the increased activity of ER*β* but not ER*α* caused by a TCS-derivative mixture, which led to neurological and behavioral abnormalities in zebrafish [87]. Also, Sprague-Dawley rats given TCS showed increased uterine weight and *Calbindin-d(9*k) (CaBP-9k) expression, which was also reciprocated in pituitary GH3 cells [178]. Reversal of both anomalies by ICI 182,780 and RU 486 points at a possible estrogenic role of the antimicrobial.

Very recently, Serra et al. [179] challenged accumulating evidence of TCS estrogenicity by showing the lack of agonistic or antagonistic effect *in vivo* and *in vitro*. While up to 0.3 *μ*M TCS did not modulate ER-dependent brain aromatase in zebrafish embryos, interference with the enzyme's activity, and with E2 activation of the enzyme observed at 1 *μ*M, was not attributed to TCS-ER interaction. Moreover, up to 10 *μ*M TCS lacked estrogenic effects in ER-expressing zebrafish liver cells as well as in MCF-7 cells [179]. Additionally, in a screening study of the estrogenicity of a group of endocrine-disrupting chemicals on fish species, TCS failed to significantly elicit a response in an *in vitro* ER*α* reporter gene assay [180].

In light of available evidence, the general consensus seems to indicate that the estrogenicity of TCS is contingent upon multiple factors, including concentration, species, duration of exposure, and whether TCS is administered alone or in combination with other molecules.

With regard to the androgenic properties of TCS, it was revealed that TCS interferes with testosterone- (TSN-) related transcription but promotes that dependent on androgen [181, 182]. In a recent *in vivo* study on weanling male rats, Riad et al. [104] reported that TCS, either alone or combined with butylparaben, reduced TSN, leutinizing hormone (LH), and follicle-stimulating hormone (FSH), while increased E2 was observed upon single TCS administration Also, TCS-induced proliferation and migration of LNCaP cells were significantly reduced in presence of bicalutamide, an androgen receptor (AR) antagonist [77]. These findings support a previous report by Ahn et al. [183] in which 1 *μ*M TCS reduced E2-induced ER activation by 50% and AR in human BG1Luc4E2 ovarian adenocarcinoma cells and T47D-ARE breast cancer cells, respectively. Evidence for TCS estrogenicity was detected in MCF7 cells when [(3)H]estradiol was successfully displaced from the ER by the antimicrobial [184]. Furthermore, 10 *μ*M TCS attenuated E2-dependent ERE-CAT reporter gene induction, while 0.1 and 1 *μ*M TCS inhibited TSN-stimulated LTR-CAT reporter gene in both T47D cells and S115 mouse mammary tumor cells [184]. TCS was also determined to have a weak effect on AhR in recombinant rat hepatoma (H4L1.1c4) cells. Finally, Forgacs et al. [185] showed that TCS interferes with recombinant hCG stimulation of TSN in a novel BLTK1 murine Leydig cell model. Most recently, however, no significant influence on androgen synthesis or activity by TCS was observed in Wistar rats [186].

Controversy surrounding the interaction between TCS and members of the peroxisome proliferator-activated receptors (PPARs) has gained considerable attention as of late. This has essentially stemmed from the apparent discrepancy between data obtained from humans and those from rodents. In comparing the differential modulation of TCS on PPAR*α* in HepG2 cells and mouse hepatoma Hepa1c1c7 cells, distinct responses were observed by Wu et al. [187]. Protein levels of PPAR*α* downstream target, acyl-coenzyme A oxidase, were decreased in HepG2 cells but were increased in Hepa1c1c7, which also showed higher DNA synthesis and blunted apoptosis through transforming growth factor (TGF-*β*). PPAR signaling was similarly identified as a target of TCS through genome-wide CRISPR-Cas9 screening in HepG2 cells [188], zebrafish [189], and *Gallus gallus* chicken embryos [190]. In the latter model, PPAR signaling members *Cyp7a1*, fatty acid-binding protein 1 (*Fabp1*), acyl-CoA synthetase long-chain family member 5 (*Acsl5*), acyl-CoA oxidase 2 (*Acox2*), and perilipin 1 (*Plin1*) were upregulated, whereas angiopoietin-like 4 (*Angptl*) was downregulated.

TCS administered to pregnant mice caused insulin resistance, hypothyroidism, diminished glucose transporter 4 (GLUT4) expression, and inhibition of Akt and mTOR phosphorylation [191, 192]. While thyroxine corrected these adversaries, PPAR*γ* activator, rosiglitazone, solely reversed the decrease in Akt phosphorylation in adipose tissue and in muscle [192]. PPAR*γ* is known to ameliorate mTOR suppression-induced glucose intolerance in rats [193], further underlining the far-reaching effects of TCS action.

Although TCS has been reported to promote hepatocyte proliferation in mice through PPAR [12], Yueh et al. [103] found no appreciable induction of PPAR*α* following TCS treatment. Importantly, the authors also identified constitutive androstane receptor (CAR) as a possible aggravator of TCS-induced tumorigenesis, given the halved tumor number in *Car*^–/−^ mice compared to their *Car*^+/−^ counterparts. TCS, as is the case with PPARs, is reported to exhibit varying affinities for CAR and pregnane X receptor (PXR) in humans and rodents. A weak agonist for human CAR, TCS was found to be a reverse agonist for rodent CAR, an agonist for human PXR, and had no effect on rodent PXR [194].

Calcium concentration within cells influences protein conformation and dynamics. Protein binding of Ca^2+^, on the other hand, maintains the ion's content within a physiological range and sets forth diverse cellular activities related to gene expression, motility, secretion, and survival [195]. Beside proteins, intracellular Ca^2+^ levels are modulated by a variety of stimuli, including xenobiotic exposure. Through the Ca^2+^ channel ryanodine (Ry) receptor type 1 (RyR1), TCS increased cytosolic Ca^2+^ dose-dependently in primary skeletal myotubes irrespective of extracellular Ca^2+^ [183]. Accordingly, muscle contractility was compromised upon TCS exposure *in vitro* and *in vivo* [196]. Results from this study indicate that TCS impaired excitation-contraction coupling (ECC) in cardiac and skeletal muscles and enhanced electrically induced Ca^2+^ transients in myotubes without depleting intracellular Ca^2+^ and notwithstanding RyR1 blockage. TCS also efficiently blocked excitation-coupled Ca^2+^ entry and interfered with the bidirectional signaling between RyR1 channels and Ca^2+^ ions. Likewise, TCS compromised ECC in larval fathead minnows *Pimephales promelas*, as evidenced by altered RyR and dihydropyridine receptor (DHPR) mRNA and protein levels and weakened ligand binding to both receptors in adult muscle homogenates [197].

In rat thymocytes, TCS elevated intracellular Ca^2+^ levels and opened Ca^2+^-responsive K^+^ channels, eventually leading to membrane hyperpolarization [198]. Also, TCS prevented Ca^2+^-induced mitochondrial swelling in rat liver [199]. A more in-depth analysis of TCS modulation of Ca^2+^ homeostasis was conducted on rat basophilic leukemia (RBL) mast cells [24]. In this cell type, TCS caused mitochondrial fission and diminished membrane potential and translocation, with compromised ATP production and elevated ROS. These changes were associated with perturbed mitochondrial and endoplasmic reticulum Ca^2+^ and depleted cytosolic Ca^2+^ levels following antigen stimulation. Accordingly, TCS-induced degranulation of mast cell may at least in part be attributed to Ca^2+^ mobilization.

Calcium modulation by TCS has also been investigated in other organisms. In *C. reinhardtii* exposed to 14 *μ*M TCS, increased Ca^2+^ levels with oxidative stress, cell and mitochondrial membrane depolarization, compromised photosynthesis, and caspase activation were noted [90]. Importantly, chelation of intracellular Ca^2+^ ions by BAPTA-AM protected the algae from TCS-induced Ca^2+^ dysregulation. These observations strongly implicate Ca^2+^ as a mediator of a wide array of toxic anomalies attributed to TCS.

Literature concerning the xenobiotic response to TCS has revealed important signaling pathways activated or suppressed by TCS (Table 6). Distinct outcomes exist among species and even within the same species based on experimental conditions and model under investigation. Although important milestones in TCS signaling have been achieved so far, there remains a lot to be discovered, especially in human-based systems, about the modulatory effects of TCS on cellular physiology. In particular, the response of many human cell types and tissues to TCS treatment is unknown, and identification of signaling pathways and their roles in cellular growth, metabolism, and overall function is therefore advised.

## 8. Therapeutic Proposals

The first specific action mechanism of TCS in prokaryotes was only demonstrated 20 years ago, when inhibition of fatty acid synthesis in *Escherichia coli* was noted following exposure to TCS [200, 201]. TCS irreversibly inhibited the fatty acid biosynthesis enzyme, enoyl–acyl carrier protein reductase (ACP), by mimicking its natural substrate *in vivo*. Further, a mutated or overexpressed ACP, encoded by *fabI*, was shown to confer TCS resistance in the bacterium. These findings established ACP as a specific, subcellular TCS target. Efforts have thus far revealed the susceptibility of a host of other pathogens to inhibition of fatty acid synthesis by TCS. These include *Staphylococcus aureus*, *M. tuberculosis*, *Helicobacter pylori*, *Haemophilus influenzae*, *Plasmodium falciparum*, *Toxoplasma gondii*, *Leishmania* spp., and *Trypanosoma* spp. [52, 202–208]. In humans, fatty acid synthase (FAS) is the only multienzyme complex that is responsible for the endogenous synthesis of saturated fatty acids from acetyl-CoA and malonyl-CoA [209, 210]. Although a BLAST analysis of *E. coli* FabI protein and FAS showed no homology, appreciable sequence similarities were nevertheless found with polyketide synthase and type I FAS of *M. tuberculosis* [211].

The success of cerulenin, a mycotoxin with fatty acid inhibitory action, in suppressing tumor progression *in vivo* has spawned several reports in support of fatty acid synthesis inhibition as an emerging target for chemotherapy [212]. The earliest study in this regard investigated the cytotoxicity of TCS in MCF-7 and SKBr-3 breast cancer cells [211]. It was revealed that TCS at 10-50 *μ*M is cytotoxic and antiproliferative, induces morphological alterations, and inhibits FAS. These findings corroborate an earlier observation linking FAS inhibition with apoptotic death of breast cancer cells [211, 213, 214]. TCS was similarly found to inhibit the development of methylnitrosourea-induced breast cancer in Sprague-Dawley rats [209]. In human A-375 melanoma cells, TCS inhibited growth at 40 *μ*M [215]. TCS was similarly found to be dose-dependently proapoptotic in prostate cancer cells, with IC_50_ values as low as 4.5-7.8 *μ*M [216]. Whereas no cytotoxicity was observed in NIH3T3 fibroblasts at concentrations up to 60 *μ*M, values of IC_50_ ranging from 0.74 to 62 *μ*M were nonetheless observed in nonmalignant prostate cells. This suggests two things; first, that prostate cells are relatively more sensitive to TCS toxicity than fibroblasts and presumably other nonmalignant cell types, and second, that malignant prostate cells exhibit higher chemosensitivity compared to their nonmalignant counterparts. This differential susceptibility could be due to overexpressed FAS in malignant cells. However, in contrast to these reports, at concentrations up to 345 *μ*M, TCS was found to be preferentially cytotoxic to Y79 RB cells over mouse 3T3 fibroblasts and human MIO-M1 Müller glial cells as indicated by IC_50_ values, creating a large therapeutic index of 7.1 and 5.3, respectively [217]. FAS suppression, depleted fatty acid content, lipid peroxidation, and apoptotic death were noted in Y79 RB cells at the same TCS concentration range [98]. Recently, TCS at 40 *μ*M was also shown to be effective against MiaPaCa-2 and AsPC-1 pancreatic cancer cells suppressing proliferation and eliciting apoptotic death [218]. Of note, in a related study, TCS impeded mouse preadipocyte differentiation [219]. Given the regulation of food intake by FAS, and the susceptibility of adipocyte development to TCS inhibition, it was suggested that TCS may possess anti-obesogenic properties.

The differential expression and activity of FAS in healthy and malignant tissues, where it is upregulated in the latter [220, 221], indicate a possibly high therapeutic index. The long history of human use, and the ubiquity of TCS in consumer products, coupled with encouraging *in vivo* results, cements the antimicrobial as a promising candidate for chemotherapy. As noted earlier, it must be stressed that variations in the final outcome of TCS treatment largely depend on experimental setup. Moreover, limited data from animal studies suggest that in the presence of a preexisting tumor, TCS administration seems to exacerbate the condition. This observation is concerning and indeed warrants further investigation before TCS can be invested in for clinical trials.

## 9. Conclusion

TCS is a synthetic antimicrobial with a long history of human use. At concentrations well below those present in commercial products, data from *in vitro* and *in vivo* studies have provided evidence of adverse effects on diverse molecular pathways. Most alarmingly is TCS enhancement of malignant cell proliferation *in vitro* and tumor growth *in vivo*. On the other hand, TCS has also been shown to be protective against malignant cell growth and proliferation, possibly opening the door for its use in chemotherapy. Clearly, dose and time dependence is an important factor in determining the eventual denouement of the chemical. In spite of the numerous publications dissecting the signaling pathways responsive to TCS, it is evident that a severe paucity surrounding human-based *in vivo* and *in vitro* studies still remains today. Future studies, thus, should focus on identifying signaling molecules differentially regulated by TCS and characterize their roles in toxic or protective effects in different cell types. Insights gained from such revelations will be invaluable to possibly validate targets for drug development or devise possible TCS adjuvants or inhibitors.

## Figures and Tables

**Table 1:** Summary of membrane and cytoskeletal targets of TCS.

Model	Target		Response
	Gene/protein	Molecular identity	
*K. pneumoniae*	*KpnGH*	Efflux pump	Sensitive to TCS

*E. coli*	*AcrAB*	Efflux pumps	Upregulated by TCS
	*acrE*		Upregulated by TCS
	*mdtE*		Upregulated by TCS
	*acrF*		Upregulated by TCS
	*mdtB*		Upregulated by TCS
	*mdtC*		Upregulated by TCS
	*yddA*		Upregulated by TCS
	*emrA*		Upregulated by TCS
	*emrE*		Upregulated by TCS
	*sanA*	Cell wall/membrane structure	Upregulated by TCS
	*dacB*		Upregulated by TCS
	*amiC*	Cell envelope	Upregulated by TCS
	*clsA*		Upregulated by TCS
	*ompX*	Membrane porin	Downregulated by TCS
	*motA*	Flagellar	Upregulated by TCS
	*flgM*		Upregulated by TCS

*R. rubrum* S1H	*sugE*	Small multidrug resistance protein	Upregulated by TCS
	*mexF*	RND efflux system, inner membrane transporter	Upregulated by TCS
	*mexB*		Upregulated by TCS
	*mexE*	RND efflux system, membrane fusion proteins	Sensitive to TCS
	*mexA*		Upregulated by TCS
	*mexM*		Upregulated by TCS
	*oprM*	RND efflux system, outer membrane transporter	Upregulated by TCS
	*glmM*	Cell envelope; phosphoglucosamine mutase	Upregulated by TCS
	*exoD*	Cell envelope; exopolysaccharide synthesis protein D	Upregulated by TCS
	*wbpM*	Cell envelope; polysaccharide biosynthesis protein M	Upregulated by TCS

*A. tumefaciens* C58	*AcrA*	RND efflux system, periplasmic adaptor protein	Upregulated by TCS

Human erythrocytes	Na^+^,K^+^,Mg^2+^-ATPase	Membrane ion transporter	Sensitive to TCS

*C. elegans*	*Pmp-3*	Membrane ABC transporter	Downregulated by TCS

*D. rerio*	Actin, cytoplasmic 2	Cytoskeleton	Downregulated by TCS
	Actin *α*1, skeletal muscle		Downregulated by TCS
	Light polypeptide 3		Downregulated by TCS
	*Desmin*	Cytoskeleton; muscular filament structure	Upregulated by TCS
	Fast skeletal muscle myosin		Sensitive to TCS
	Keratin, type I cytoskeletal 18		Upregulated by TCS
	Tropomyosin *α*-1 chain		Downregulated by TCS
	Type II cytokeratin		Upregulated by TCS
	Lamin B1	Cytoskeleton; nuclear lamina	Downregulated by TCS

*D. polymorpha*	Tubulin *β*-2/*α*-4 chain	Cytoskeleton	Upregulated by TCS
	Tubulin *β*-4 chain		Upregulated by TCS
	Myosin light chain	Cytoskeleton; muscular filament structure	Upregulated by TCS

Abbreviation: RND: resistance-nodulation-division; ABC: ATP-binding cassette.

**Table 2:** Summary of cell survival molecules modulated by TCS.

Model	Target		Response
	Gene/protein	Molecular identity	
JEG-3 cells	Estradiol	Major female sex hormones	Upregulated by TCS
	Progesterone		Upregulated by TCS
	*β*-hCG	Maintenance of pregnancy	Downregulated by TCS
	Caspase-3	Apoptosis regulator; proapoptotic	Upregulated by TCS

BG-1 cells	Cyclin D1	Cell cycle regulators	Upregulated by TCS
	p21		Downregulated by TCS
	Bax	Apoptosis regulator; proapoptotic	Downregulated by TCS

MCF-7 cells	Cyclin D1	Cell cycle regulators	Upregulated by TCS
	Cyclin E		Upregulated by TCS
	p21		Downregulated by TCS
	Bax	Apoptosis regulator; proapoptotic	Downregulated by TCS
	Cathepsin B	Metastasis markers	Upregulated by TCS
	Cathepsin D		Upregulated by TCS
	MMP-9		Upregulated by TCS
	MMP-2		Upregulated by TCS
	CXCR4		Upregulated by TCS
	Snail	Mesenchymal markers	Upregulated by TCS
	Slug		Upregulated by TCS

LNCaP	p21	Cell cycle regulator	Downregulated by TCS

Primary human syncytiotrophoblasts	11*β*-HSD2	Fetal development; anticortisol	Downregulated by TCS
	Caspase-3	Apoptosis regulators; proapoptotic	Upregulated by TCS
	Bax		Upregulated by TCS
	Bcl-2	Apoptosis regulator; antiapoptotic	Downregulated by TCS

Rat neural stem cells	Caspase-3	Apoptosis regulators; proapoptotic	Upregulated by TCS
	Bax		Upregulated by TCS
	Bcl-2	Apoptosis regulator; antiapoptotic	Downregulated by TCS

Mouse neocortical neurons	*GluN1*	Ionotropic glutamate receptors; neurotransmission	Downregulated by TCS
	GluN1		Downregulated by TCS
	*GluN2A*		Downregulated by TCS
	GluN2A		Downregulated by TCS
	*GluN2B*		Upregulated by TCS
	GluN2B		Downregulated by TCS
	FasR	Apoptosis regulators; proapoptotic	Upregulated by TCS
	Caspase-8		Upregulated by TCS
	Caspase-9		Upregulated by TCS
	Caspase-3		Upregulated by TCS
	AhR	Ligand-activated receptor; detoxification	Upregulated by TCS

JB6 Cl 41-5a cells	Cyclin D1	Cell cycle regulators	Upregulated by TCS
	Cyclin A		Upregulated by TCS
	p27		Downregulated by TCS

B6C3F1 mice	*Alp*	Pluripotency markers; stem cell self-renewal and differentiation regulators	Downregulated by TCS
	*Oct4*		Downregulated by TCS
	*Nanog*		Downregulated by TCS
	ALP		Downregulated by TCS
	Oct 4		Downregulated by TCS
	Nanog		Downregulated by TCS
	Sox 2		Downregulated by TCS
	miRNA-134	Transcriptional regulator of pluripotency markers	Upregulated by TCS

*D. rerio*	*Oct4*	Pluripotency markers	Downregulated by TCS
	*Nanog*		Downregulated by TCS
	*Sox2*		Upregulated by TCS
	*p53*	Cell cycle regulator; tumor suppressor	Upregulated by TCS
	*Casp3*	Apoptosis regulators; proapoptotic	Upregulated by TCS
	*Casp8*		Upregulated by TCS
	*Shha*	Early neurogenesis	Sensitive to TCS sensitive to TCS
	*Ngn1*		Upregulated by TCS
	*Nrd*		Upregulated by TCS
	*Elavl3*		Upregulated by TCS
	*α1-tubulin*	Neural maturation	Upregulated by TCS
	*Gap43*		Upregulated by TCS
	*Gfap*		Downregulated by TCS
	*Mbp*		Downregulated by TCS

Abbreviation: *Shha*: sonic hedgehog a; *Ngn1*: neurogenin 1; *Nrd*: NeuroD; *Elavl3*: ELAV-like, neuron-specific RNA-binding protein 3; *Gap43*: growth-associated protein 43; *Gfap*: glial fibrillary acidic protein; *Mbp*: myelin basic protein.

**Table 3:** Oxidative stress patterns elicited by TCS.

Model	Target		Response
	Biomarker	Molecular identity	
Humans (pregnant women; children)	Urinary 8-OHdG	Oxidized deoxyguanosine; DNA damage	Upregulated by TCS

Nthy-ori 3-1 cells	ROS	Metabolic oxygen by-products	Upregulated by TCS
PBMC^∗^	ROS		Upregulated by TCS
	Lipid peroxidation	Oxidized lipids	Upregulated by TCS
	Protein carbonylation	Oxidized proteins	Upregulated by TCS

Y79 RB cells	Lipid peroxidation	Oxidized lipids	Upregulated by TCS

Human bone marrow-derived mesenchymal stem cells	Nrf2	Antioxidant regulator	Downregulated by TCS
	*Ho-1*	Antioxidant enzymes	Downregulated by TCS
	*Nqo-1*		Downregulated by TCS

VM7Luc4E2 cells	ROS	Metabolic oxygen by-products	Upregulated by TCS

Mouse liver	O_2_^–^	Antioxidant enzymes	Upregulated by TCS
	HO-1		Upregulated by TCS
	NQO-1		Upregulated by TCS
	GST		Upregulated by TCS

Weanling rats	MDA	Oxidized lipid marker	Upregulated by TCS
	SOD	Antioxidant enzymes	Upregulated by TCS
	CAT		Downregulated by TCS

Female albino rat lung homogenates	Lipid peroxidation	Oxidized lipids	Upregulated by TCS
	SOD	Antioxidants	Downregulated by TCS
	CAT		Downregulated by TCS
	GSH		Downregulated by TCS

C57BL/6 mice liver	*Gpx1*	Antioxidant enzyme; glutathione homeostasis	Upregulated by TCS
	*Aox1*	Superoxide and hydrogen peroxide formation	Upregulated by TCS

Sprague-Dawley rat hypothalamus	MDA	Oxidized lipid marker	Upregulated by TCS
	*Gpx3*	Antioxidant enzyme; glutathione homeostasis	Downregulated by TCS
	*Cat*	Antioxidant enzymes	Downregulated by TCS
	*Sod2*		Downregulated by TCS

Rat neural stem cells	ROS	Metabolic oxygen by-products	Upregulated by TCS
	GSH	Antioxidant	Downregulated by TCS

Mouse neocortical neurons	ROS	Metabolic oxygen by-products	Upregulated by TCS
	*Cyp1a1*	Cytochrome family enzymes; detoxification	Downregulated by TCS
	CYP1a1		Downregulated by TCS
	*Cyp1b1*		Downregulated by TCS
	Cyp1b1		Upregulated by TCS
Sprague-Dawley rat liver	*Cyp1a1*		Upregulated by TCS
	*Cyp1a2*		Upregulated by TCS
	*Cyp2b1*		Upregulated by TCS
	CYP2b1		Upregulated by TCS
	*Cyp3a1*		Upregulated by TCS
	*Ugt2b1*	Glucuronidation enzymes; detoxification	Upregulated by TCS
	Ugt2b1		Upregulated by TCS
	*Sult1e1*	Sulfation enzyme; detoxification	Upregulated by TCS
	Sult1e1		Upregulated by TCS

*C. elegans*	ROS	Metabolic oxygen by-products	Upregulated by TCS
	*Skn1*	Stress response regulator	Upregulated by TCS
	SKN-1		Downregulated by TCS
	*Gcs1*	Antioxidant enzymes	Downregulated by TCS
	*Sod1*		Upregulated by TCS
	*Sod4*		Upregulated by TCS
	*Hsp-3*	Stress response; protein stabilization	Upregulated by TCS
	*Hsp-4*		Upregulated by TCS
	*Hsp-16.2*		Upregulated by TCS
	*Hsp-70*		Upregulated by TCS
	*Cyp29A2*	Cytochrome family enzymes; detoxification	Upregulated by TCS
	*Cyp34A9*		Upregulated by TCS
	DAF-16	Stress response	Upregulated by TCS

*E. fetida*	MDA	Oxidized lipid marker	Upregulated by TCS
	CAT	Antioxidant enzymes	Upregulated by TCS
	GST		Upregulated by TCS
	SOD		Sensitive to TCS to TCS

*A. fulica*	MDA	Oxidized lipid marker	Upregulated by TCS
	CAT	Antioxidant enzymes	Sensitive to TCS to TCS
	SOD		Downregulated by TCS
	POD		Downregulated by TCS

ZFL liver cells	CYP1A	Cytochrome family enzyme; detoxification	Upregulated by TCS

*D. rerio* larvae	GPx	Antioxidant enzymes; glutathione homeostasis	Upregulated by TCS
	GR		Downregulated by TCS
	PRD-2	Antioxidant enzyme	Downregulated by TCS
	Hsp-5	Stress response; protein stabilization	Upregulated by TCS
	Hsp-90 *β*		Upregulated by TCS

*D. magna*	MDA	Oxidized lipid marker	Upregulated by TCS
	CAT	Antioxidant enzymes	Sensitive to TCS to TCS
	EROD	Detoxification enzymes	Sensitive to TCS to TCS
	ERND		Sensitive to TCS to TCS
	APND		Sensitive to TCS to TCS
	Glutamine	Amino acids; markers of protein oxidation/breakdown	Upregulated by TCS
	Glutamate		Upregulated by TCS
	Proline		Upregulated by TCS

*D. polymorpha* gills	Hsp-70	Stress response; protein stabilization	Sensitive to TCS to TCS

*U. tumidus* digestive gland	GAPDH	Oxidoreductase; glucose metabolism	Sensitive to TCS to TCS
	GSSG	Oxidized glutathione; antioxidant	Upregulated by TCS
	Oxyradicals	Oxygen-containing radicals; prooxidants	Downregulated by TCS
	Lipofuscin	Lysosomal pigment granules; toxicity marker	Downregulated by TCS

*T. japonicus*	ROS	Metabolic oxygen by-products	Upregulated by TCS
	*Sod*	Antioxidant enzymes	Sensitive to TCS to TCS
	SOD		Upregulated by TCS
	*Cat*		Sensitive to TCS to TCS
	*Gst* variants	Antioxidants; glutathione homeostasis	Sensitive to TCS to TCS
	GST		Upregulated by TCS
	GPx		Upregulated by TCS
	GSH		Upregulated by TCS
	*Cyp3026a3*	Cytochrome family enzymes; detoxification	Upregulated by TCS
	*Cyp3037a1*		Upregulated by TCS

*P. fulvidraco*	MDA	Oxidized lipid marker	Sensitive to TCS to TCS
	CAT	Antioxidant enzyme	Upregulated by TCS
	*Gst*	Antioxidant enzyme; glutathione homeostasis	Sensitive to TCS to TCS
	EROD	Detoxification enzymes	Upregulated by TCS
	ERND		Upregulated by TCS
	APND		Upregulated by TCS
	*Cyp1a*	Cytochrome family enzymes; detoxification	Sensitive to TCS to TCS
	*Cyp3a*		Sensitive to TCS to TCS

*H. fossilis*	CAT	Antioxidant enzymes	Upregulated by TCS
	SOD		Upregulated by TCS
	GSH	Antioxidants; glutathione homeostasis	Downregulated by TCS
	GST		Downregulated by TCS
	GPx		Downregulated by TCS

*C. auratus*	MDA	Oxidized lipid marker	Upregulated by TCS
	CAT	Antioxidant enzymes	Upregulated by TCS
	SOD		Downregulated by TCS
	GSH	Antioxidant; glutathione homeostasis	Upregulated by TCS

*Brachionus koreanus*	ROS	Metabolic oxygen by-products	Upregulated by TCS
	*Gst* variants	Antioxidant enzyme; glutathione homeostasis	Sensitive to TCS to TCS
	*Gpx*		Sensitive to TCS to TCS
	GST		Upregulated by TCS
	*Sod*	Antioxidant enzymes	Sensitive to TCS to TCS
	*Cat*		Sensitive to TCS to TCS
	*Cyp3042a1*	Cytochrome family enzymes; detoxification	Sensitive to TCS to TCS
	*Cyp43a1*		Sensitive to TCS to TCS
	*Hsp10*	Stress response; protein stabilization	Sensitive to TCS to TCS
	*Hsp21*		Upregulated by TCS
	*Hsp27*		Upregulated by TCS
	*Hsp30*		Sensitive to TCS to TCS
	*Hsp40*		Sensitive to TCS to TCS
	*Hsp40h*		Sensitive to TCS to TCS
	*Hsp60*		Sensitive to TCS to TCS
	*Hsp70*		Upregulated by TCS
	*Hsc70*		Upregulated by TCS
	*Hsp90α1*		Sensitive to TCS to TCS
	*Hsp90α2*		Sensitive to TCS to TCS
	*Hsp90β*		Sensitive to TCS to TCS

*B. gargarizans* liver	*Sod*	Antioxidant enzyme	Downregulated by TCS
	*Phgpx*	Antioxidant enzyme; glutathione homeostasis	Downregulated by TCS
*P. perezi* larvae	GST		Upregulated by TCS

*R. philippinarum* digestive gland	MDA	Oxidized lipid marker	Upregulated by TCS
	CAT	Antioxidant enzymes	Sensitive to TCS to TCS
	SOD		Sensitive to TCS to TCS
	GPx variants	Antioxidant enzymes; glutathione homeostasis	Sensitive to TCS to TCS
	GST		Sensitive to TCS to TCS
	GR		Sensitive to TCS to TCS
	EROD	Detoxification enzyme	Sensitive to TCS to TCS

*O. mykiss* liver and kidney	*Cat*	Antioxidant enzymes	Downregulated by TCS
	*Sod*		Upregulated by TCS
	*Gpx* variants	Antioxidant enzymes; glutathione homeostasis	Upregulated by TCS
	*Gsta*		Upregulated by TCS
	*Hsp90bb*	Stress response; protein stabilization	Upregulated by TCS
	*Hsp90ba*		Upregulated by TCS
	*Hsc70a*		Upregulated by TCS

*C. reinhardtii*	ROS	Metabolic oxygen by-products	Upregulated by TCS
	MDA	Oxidized lipid marker	Upregulated by TCS
	*Sod*	Antioxidant enzyme	Upregulated by TCS
	*Gpx*	Antioxidant enzyme; glutathione homeostasis	Upregulated by TCS
*R. rubrum* S1H	*Gpx*		Upregulated by TCS
	*GrxC*	Antioxidant enzymes; glutathione homeostasis	Upregulated by TCS
	*TrxB*		Upregulated by TCS
	*OsmC*	Antioxidant enzyme	Upregulated by TCS
	*DnaJ*	Heat shock protein; general stress marker	Upregulated by TCS
	*RpoN*	RNA polymerase factor sigma-54; general stress marker	Downregulated by TCS
	*TerA*	Tellurite resistance protein A; general stress marker	Upregulated by TCS
	*Psp* variants	Phage shock proteins; general stress markers	Sensitive to TCS to TCS
	*ClpP*	ATP-dependent protease, proteolytic subunit; general stress marker	Upregulated by TCS
	*HrcA*	Heat-inducible transcription suppressor; general stress marker	Upregulated by TCS

*E. coli* K12, MG1655	*OxyR*	ROS sensor proteins	Upregulated by TCS
	*Grx*	Antioxidant enzymes; glutathione homeostasis	Upregulated by TCS
	*Sod* variants	Antioxidant enzymes	Upregulated by TCS
	*Cat* variants		Upregulated by TCS
	*Ahp* variants	Antioxidant enzymes	Upregulated by TCS

*E. coli*	ROS	Metabolic oxygen by-products	Upregulated by TCS
	*YgiW*	Antioxidant proteins	Downregulated by TCS
	*SoxS*		Downregulated by TCS
	*YhcN*		Downregulated by TCS

Abbreviation: *TrxB*: thioredoxin; *OsmC*: peroxiredoxin osmotically inducible protein C-like. ^∗^Effects of 2,4-DCP, a by-product of TCS degradation.

**Table 4:** Inflammatory and immune mediators responsive to TCS.

Model	Target		Response
	Biomarker	Molecular identity	
Human gingival fibroblasts	COX-1/2	Inflammatory mediators	Downregulated by TCS
	5/15-LPO		Downregulated by TCS
	PGE2		Downregulated by TCS
	PGI2		Downregulated by TCS
	Arachidonic acid		Downregulated by TCS
	PLA2		Downregulated by TCS
	PGE synthase-1		Downregulated by TCS
	IFN*γ*	Immune/inflammatory cytokines	Downregulated by TCS
	IL-1*β*		Downregulated by TCS
	MHC II	Cell surface proteins; adaptive immunity regulators	Downregulated by TCS
	*Cox2*	Inflammatory mediator	Downregulated by TCS
	*Il6*	Immune/inflammatory cytokines	Downregulated by TCS
	*Il1b*		Downregulated by TCS
	*Tlr6*	Innate immunity receptor	Upregulated by TCS

Human primary oral epithelial cells	IL-8	Immune/inflammatory cytokines	Downregulated by TCS
	IL-1*α*		Downregulated by TCS
	TNF*α*		Downregulated by TCS
	miR146a	Transcriptional regulators of TLR response	Upregulated by TCS
	miR155s		Downregulated by TCS

Mouse skin and leukocytes	S100A8/A9	Inflammatory modulator; Ca^2+^-binding protein	Upregulated by TCS
	*Tlr4*	Innate immunity receptors	Upregulated by TCS
	TLR4		Upregulated by TCS
	*Tlr1*		Upregulated by TCS
	*Tlr2*		Upregulated by TCS
	*Tlr6*		Upregulated by TCS

Rat osteoblastic osteosarcoma cells	MMP-13	Endopeptidase; collagen degradation	Downregulated by TCS

Human oral fluids	IL-1*α*	Immune/inflammatory cytokines	Downregulated by TCS
	IL-1*β*		Sensitive to TCS
	IL-8		Sensitive to TCS
	MCP-1		Sensitive to TCS
	TIMP-2	MMP regulator proteins	Sensitive to TCS
	TIMP-1		Downregulated by TCS
	MMP-8/9	Endopeptidases; extracellular matrix degradation	Downregulated by TCS

Human urine	IL-6	Immune/inflammatory cytokines	Upregulated by TCS
Sprague-Dawley rats	TNF*α*		Upregulated by TCS
	IL-6		Upregulated by TCS

Human whole blood leukocytes	*Csf2*	Hematopoietic stem cell growth and maintenance	Downregulated by TCS
	*Ifna1*	Immune/inflammatory cytokines	Downregulated by TCS
	*Ifna2*		Downregulated by TCS
	*Ifna4*		Downregulated by TCS
	*Ifna8*		Downregulated by TCS
	*Il-1f10*		Downregulated by TCS
	*Il-1f5*		Downregulated by TCS
	*Il-1f7*		Downregulated by TCS
	*Il-1f8*		Downregulated by TCS
	*Il-1f9*		Downregulated by TCS
	*Il-6*		Downregulated by TCS
	*Il-11*		Downregulated by TCS
	*Il-13*		Downregulated by TCS
	*Il-25*		Downregulated by TCS
	*Il-19*		Downregulated by TCS
	*Il-21*		Downregulated by TCS
	*Il-9*		Downregulated by TCS
	*Cd70*	Cell surface receptor/ligand; activated lymphocytes	Downregulated by TCS
	*Bmp2*	Growth factors; bone and cartilage development	Upregulated by TCS
	*Bmp6*		Upregulated by TCS
	*Tnfrsf11b*	TNFSF11 receptor	Downregulated by TCS
	*Gdf3*	Growth/differentiation factors	Downregulated by TCS
	*Gdf2*		Downregulated by TCS
	*Gdf5*		Downregulated by TCS
	*Gdf9*		Downregulated by TCS
	*Inhba*	Hypothalamus-pituitary axis regulator	Downregulated by TCS
	*Lefty2*	Left-right determination factor 2; left-right asymmetry of organs	Downregulated by TCS

Sprague-Dawley rats	TNF*α*	Immune/inflammatory cytokine	Upregulated by TCS
	IL-6		Upregulated by TCS

Abbreviation: MCP: monocyte chemoattractant protein; TIMP: tissue inhibitor of metalloproteinase; *Bmp*: bone morphogenetic protein; *Gdf*: growth differentiation factor; *Inhba*: inhibin beta A chain.

**Table 5:** TCS genotoxicity and carcinogenicity.

Model	Effect	Classification
HepG2 cells	Global DNA hypomethylation	Limited evidence of carcinogenicity
V79 cells	Chromosomal aberrations	
Mouse	Somatic mutation (positive spot test) Increased incidence of liver tumors Aggravated hepatocellular carcinoma Exacerbated colon tumorigenesis	

*D. polymorpha*	DNA damage (positive comet assay)	N/A
*U. tumidus*	DNA strand breaks (Hoescht 33342 fluorescence)	
*E. Fetida*	DNA damage (positive Comet assay)	
*D. magna*	DNA damage (positive Comet assay)	
*A. salina*	DNA damage (positive Comet assay)	
*H. fossilis*	DNA damage (positive Comet assay)	
*C. auratus*	DNA damage (positive Comet assay)	
*O. mykiss*	DNA damage (positive Comet assay)	
*T. thermophila*	DNA damage (positive Comet assay)	
*A. cepa*	Chromosomal stickiness, reduced mitotic activity, and ana-telophase bridges (positive Feulgen reaction)	

N/A = data from non-mammalian animals are not considered for ECHA mutagenicity/carcinogenicity classification.

**Table 6:** TCS modulation of signaling pathways.

Model	Target		Response
	Pathways	TCS role	
H460 cells	FAK/Akt	Cellular migration and invasion	Upregulated by TCS
	Rac1		Upregulated by TCS

JB6 Cl 41-5a cells	ERK1/2	Cell proliferation	Upregulated by TCS
	JNK		Upregulated by TCS
	p38		Upregulated by TCS
	Akt		Upregulated by TCS
	PI3K		Upregulated by TCS

Rat neural stem cells	JNK	Cytotoxicity and apoptosis	Upregulated by TCS
	p38		
			Upregulated by TCS
	ERK		
	Akt		Downregulated by TCS
	PI3K		Downregulated by TCS
			Downregulated by TCS

Sprague-Dawley rats hypothalamus and Nthy-ori 3-1 cells	JNK	Reduced TPO; hypothyroidism	Upregulated by TCS
	p38		Upregulated by TCS

Whole blood leukocytes	*Nik*	Anti-inflammatory response	Downregulated by TCS
	*Cjun*		Downregulated by TCS
Mouse osteoblastic osteocarcinoma	*Fos*		Downregulated by TCS
	*Jun*		Downregulated by TCS
	*Ap1*		Downregulated by TCS

BG-1	ER*α*	Cell proliferation	Upregulated by TCS
MCF-7 cells	ER*α*^∗^		Sensitive to TCS
	pIRS-1		Upregulated by TCS
	pAKT		Upregulated by TCS
	pMEK1/2		Upregulated by TCS
	pERK1/2		Upregulated by TCS
VM7Luc4E2 cells	*Erα*		Downregulated by TCS
	*Ps2*		Upregulated by TCS
	ER*α*		Downregulated by TCS
	pS2		Upregulated by TCS
	miR-22		Upregulated by TCS
	miR-206		Upregulated by TCS
	miR-193b		Upregulated by TCS

Sheep placenta	E2	Anti-estrogenicity	Downregulated by TCS
	Estrogen sulfotransferase		Downregulated by TCS
BG1Luc4E2 cells	ER^∗^		Downregulated by TCS

Sprague-Dawley rats and GH3 cells	*CaBP-9* k	Estrogenicity	Upregulated by TCS

LNCaP	AR	Androgenicity; cell proliferation, and migration	Upregulated by TCS

T47D-ARE cells	AR	Anti-androgenicity	Downregulated by TCS

H4L1.1c4 cells	AR	Pro(anti)-androgenicity	Sensitive to TCS

HepG2 cells	Acyl-coenzyme A oxidase	Blunted lipid metabolism	Downregulated by TCS

Hepa1c1c7 cells	Acyl-coenzyme A oxidase	Enhanced lipid metabolism and DNA synthesis	Upregulated by TCS
	TGF-*β*	Antiapoptosis	Downregulated by TCS

*D. rerio*	PPAR*α*	Enhanced lipid metabolism	Upregulated by TCS
	PPAR*γ*		Upregulated by TCS
*G. gallus* embryo livers	PPAR*α*		Upregulated by TCS

ICR mice	Akt	Impaired glucose metabolism	Downregulated by TCS
	mTOR		Downregulated by TCS

C57BL/6 mice	CAR	Tumorigenesis	Upregulated by TCS

HepG2 cells	CAR	Enhanced hepatic catabolism	Upregulated by TCS
	PXR		Upregulated by TCS

Rodent FAO hepatoma cells	CAR	Reduced hepatic catabolism	Downregulated by TCS

Primary skeletal myotubes	Ca^2+^	Diminished muscle contractility	Upregulated by TCS
	RyR1		Upregulated by TCS
*P. promelas* muscle homogenates	*Ryr2*		Sensitive to TCS
	*Ryr3*		Downregulated by TCS
	RyR		Downregulated by TCS

Rat thymocytes	Ca^2+^	Cell membrane hyperpolarization	Upregulated by TCS

RBL cells	Ca^2+^	Mast cell degranulation	Downregulated by TCS

*C. reinhardtii*	Ca^2+^	Dampened photosynthesis	Upregulated by TCS

^∗^TCS is anti-estrogenic in the presence of E2.
